# The peanut *Ubiquitin4* promoter drives stable gene overexpression and efficient multiplex CRISPR/Cas9 gene editing in peanut

**DOI:** 10.1007/s42994-025-00230-7

**Published:** 2025-07-16

**Authors:** Yuanyuan Cui, Qianqian Zhang, Qingjing Meng, Xiaoyu Liu, Xiaoqin Liu

**Affiliations:** Peking University Institute of Advanced Agicultural Sciences, Shandong Laboratory for Advanced Agicultural Sciences at Weifang, Weifang, 261325 China

**Keywords:** Peanut, Ubiquitin promoter, Genetic transformation

## Abstract

The development of effective genetic transformation tools is crucial for advancing molecular breeding in peanut (*Arachis hypogaea*). In this study, we identified and characterized a *Ubiquitin* (*UBQ*) promoter from peanut and evaluated its potential utility in transgenic research. Using sequence similarity–based identification and transcriptome analysis, we selected a highly expressed *UBQ* gene, arahy.E356RC, designated *AhUBQ4*, from which we cloned a 973-bp fragment of the promoter region. To assess its activity, we used this *AhUBQ4* promoter fragment to drive expression of the *GUS* and *Ruby* reporter genes in transient and stable expression assays in *Nicotiana benthamiana* and peanut tissues. Compared to the commonly used CaMV 35S promoter, the *AhUBQ4* promoter had stable and high transcriptional activity across multiple tissues. Furthermore, we replaced the traditional 35S promoter with the *AhUBQ4* promoter in a CRISPR/Cas9 system, enabling efficient gene editing in peanut. Using a peanut hairy root transformation system, we induced site-specific mutations in *HY5-HOMOLOG*, confirming stable *Cas9* expression from the *AhUBQ4* promoter for genome editing applications. Our findings highlight the potential of the *AhUBQ4* promoter as a valuable genetic tool for improving transformation efficiency and gene expression stability in peanut, paving the way for enhanced functional genomics studies and molecular breeding efforts.

**Supplementary Information:**

The online version contains supplementary material available at 10.1007/s42994-025-00230-7.

Dear Editor,

Cultivated peanut (*Arachis hypogaea*) is an important global crop and a key source of oil and protein (Hammons et al. 2016). Genetic engineering has been widely applied for improvement of crops including soybean (*Glycine max*) (Bonny 2008), maize (*Zea mays*) (Yadava et al. 2017), and cotton (*Gossypium hirsutum*) (Ge et al. 2023). However, the lack of efficient tools for stable gene expression has limited the genetic engineering of peanut (Krishna et al. 2015).

In peanut transgenic research, foreign promoters such as the cauliflower mosaic virus (CaMV) 35S promoter are commonly used (Han et al. 2023; Zhou et al. 2023). However, these promoters face issues such as lower expression, gene silencing, and methylation over several generations. For example, the DNA methylation in the *35S* promoter region leads to the silencing of the exogenous gene in the transgenic offspring of birch (*Betula platyphylla*) (Ma et al. 2020). Foreign promoters also often display uneven expression. In cotton, the 35S promoter drives high expression in cotyledon and leaf vascular tissues, whereas *GUS* expression driven by the same promoter was stronger in leaves and stems than in flowers and seeds of *Nicotiana benthamiana* plants (Malik et al. 2002; Sunilkumar et al. 2002). Moreover, foreign promoters often incur a higher regulatory burden. These challenges highlight the need for effective native promoters to ensure stable gene expression in peanut transformation.

Among native promoters, those from *Ubiquitin* (*UBQ*) genes are notable for their high transcriptional activity and low risk of methylation. Widely used across plant species, they provide strong and stable expression owing to the essential nature of ubiquitin activity (Mann et al. 2011). Plant *UBQ* promoters often outperform the 35S promoter in terms of their transcriptional output. For example, *Ubiquitin* (*Ubi*) promoters from maize (*Zea maize*) drive high expression in monocot species, contributing to efficient transformation (Christensen and Quail 1996). Similarly, the Arabidopsis (*Arabidopsis thaliana*) and apple (*Malus domestica*) *UBQ10* promoters enable strong expression of several transgenes (Wang et al. 2021). These advantages make *UBQ* promoters useful tools for stable gene expression in transgenic plants.

In legumes, strong native promoters such as *GmUBQ* in soybean and *LjUBQ* in *Lotus japonicus* have demonstrated stable performance for transgene expression and genome editing applications, often outperforming viral promoters such as the 35S promoter. For instance, several *GmUBQ* promoters showed up to sevenfold higher expression than the 35S promoter in soybean, with consistent performance across transient and stable transformation systems (Hernandez-Garcia et al. 2010). Similarly, the *LjUBQ1* promoter exhibited stronger activity than the 35S promoter in various tissues of *L. japonicus*, and was incorporated into vectors for overexpression and gene silencing (Maekawa et al. 2008). Additionally, the promoters of orthologous genes such as *Nodule autoregulation receptor kinase* (*GmNARK*) and *Hypernodulation aberrant root formation 1* (*LjHAR1*) can function interchangeably across species, indicating a degree of conservation in their regulatory elements (Nontachaiyapoom et al. 2007). However, strong native promoters for transgene in peanut remain underexplored. Considering species-specific epigenetic contexts and regulatory factors, identifying and characterizing a native peanut promoter is critical for overcoming transformation bottlenecks and improving transgene stability in this species.

In this study, we identified and characterized the promoter of a highly expressed *UBQ* gene in peanut and demonstrated its potential for peanut transformation. We validated its ability to drive gene expression through transient and stable expression assays. When we replaced the traditional 35S promoter with this peanut *UBQ* promoter in a CRISPR/Cas9 vector, we achieved efficient multiplex genome editing. These results highlight the importance of deploying native peanut promoters to improve transformation efficiency and stability, offering valuable tools for plant biotechnology and functional genomics.

Based on sequence similarity searches with Arabidopsis *UBQ* genes in the peanut genome, we identified 18 *UBQ* genes in the allotetraploid peanut genome. We examined their expression patterns across 15 different tissues in transcriptome data from public databases (Clevenger et al. 2016) (Fig. 1A). One gene, arahy.E356RC (*AhUBQ4* hereafter), exhibited much higher expression in most tissues than the other *UBQ* genes, with an average Fragments Per Kilobase of transcript per Million mapped reads (FPKM) value of 2,170, which is 4.1 times that of the second most highly expressed *AhUBQ* gene (528 FPKM). In 14 tissues, the FPKM values of *AhUBQ4* exceeded 1,000 and exceeded 3,000 in flowers and two early stages of seed development. *AhUBQ4* expression dropped sharply in mature seeds, but remained above 400 FPKM (Supplementary Table 1).

**Fig. 1:**
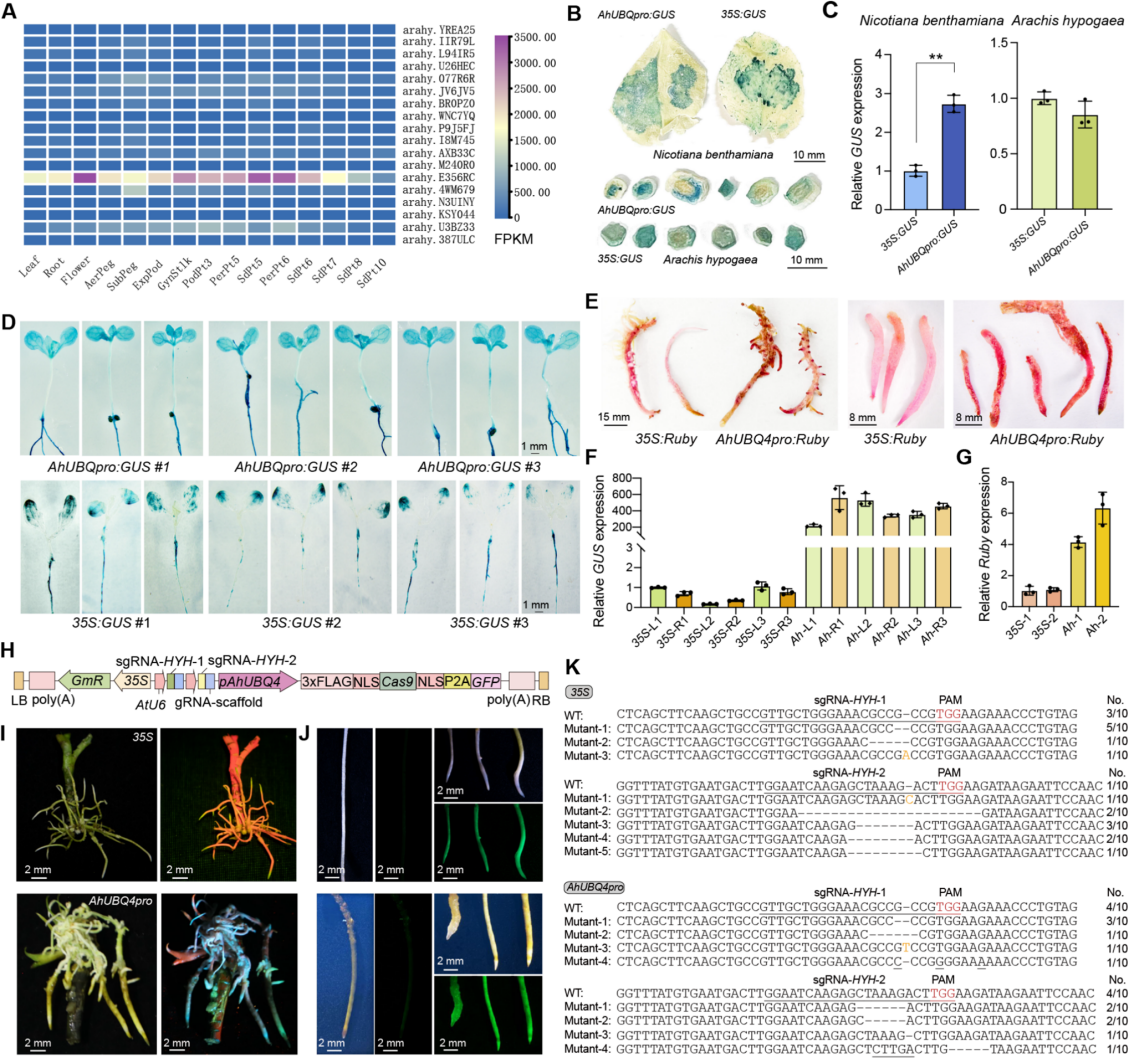
Identification and evaluation of the peanut *Ubiquitin* gene with the strongest promoter, *AhUBQ4*, via transient and stable transformation, and its application in gene editing of transgenic peanut roots. **A** Heatmap representation of expression levels for various *UBQ* genes in peanut. **B** GUS staining of *Nicotiana benthamiana* leaves (top) and peanut stem discs (bottom) infiltrated with *AhUBQ4pro:GUS* or *35S:GUS* reporter constructs. **C** Relative *GUS* expression levels in *N. benthamiana* leaves and peanut stem discs infiltrated with the *AhUBQ4pro:GUS* or *35S:GUS* reporter construct, normalized to *NbL25* (*N. benthamiana*) and *AhELF1B* (peanut) reference transcripts (see Supplementary Table 3). Data represent the mean ± standard deviation (SD) from three technical replicates. **D** GUS staining of transgenic Arabidopsis seedlings transformed with the *AhUBQ4pro:GUS* or *35S:GUS* reporter construct. **E** Ruby reporter activity in transgenic peanut hairy roots expressing *Ruby* driven by the *AhUBQ4* or 35S promoter. **F** Relative *GUS* expression levels in the stable transgenic Arabidopsis seedlings shown in (**E**). Relative *GUS* expression was measured in leaves (L) and roots (R) from three independent transgenic lines for each promoter. **G** Relative *Ruby* expression levels in transgenic peanut hairy roots shown in (**F**). Values are means ± SD from three technical replicates. **H** Diagram of the CRISPR/Cas9 vector. The construct includes a 35S promoter–driven selection marker (*GmR*), two *AtU6* promoter–driven sgRNAs (sgRNA-*HYH-1* and sgRNA-*HYH-2*), and *Cas9* placed under the control of the *AhUBQ4* promoter (replacing the 35S promoter). The *Cas9* cassette is cloned in-frame with the sequence encoding a 3 × FLAG tag, two nuclear localization signals (NLS), porcine teschovirus-1 2A peptide (P2A), and GFP, enabling gene editing and GFP fluorescence detection in transgenic tissues. **I** GFP fluorescence observation of positive transgenic roots under handheld fluorescence imaging, with the 35S promoter–driven construct (top) and the *AhUBQ4* promoter–driven construct (bottom). Left, bright-field illumination; right image, GFP fluorescence. **J** Fluorescence observation of transgenic and non-transgenic roots under a stereomicroscope. Left panels, non-transgenic roots, with bright-field (left) and GFP fluorescence (right) images confirming the absence of GFP accumulation. Right, transgenic hairy roots, with bright-field (top) and GFP fluorescence (bottom) images, demonstrating strong fluorescence in successfully transformed roots. **K** Analysis of *AhHYH* gene editing in transgenic hairy roots, with the 35S promoter–driven construct (top) and the *AhUBQ4* promoter–driven construct (bottom). Sequence alignment of the *AhHYH* target sites reveals different mutation types among edited hairy roots. The wild-type (WT) sequences are shown on top, with the protospacer-adjacent motifs (PAM) shown in red. Mutant sequences exhibit various insertions (shown in orange), deletions (−), and substitutions (underlined), with the frequency of each mutation type indicated as a fraction of total sequenced clones (No./10)

To test whether the *AhUBQ4* promoter could drive expression of other genes, we cloned 2,000-bp and 973-bp *AhUBQ4* promoter fragments upstream of the ATG (Supplementary Table 2 and Fig. S1A). To test these fragments, we infiltrated Agrobacterium (*Agrobacterium tumefaciens*) cultures harboring the reporter constructs with the *AhUBQ4* fragments, or 35S promoter driving *GUS*, followed by GUS staining of the infiltrated tissues. In *Nicotiana benthamiana* leaves, we detected uniform and intense blue staining in *AhUBQ4pro:GUS* samples with both fragments, indicating strong and consistent *GUS* expression, whereas 35S promoter–driven samples displayed patchy or uneven staining (Fig. 1B). In *N. benthamiana* leaves, the 2000-bp promoter fragment showed higher *GUS* expression. In peanut stem discs, the 973-bp promoter fragment showed higher *GUS* expression (Fig. S1B) and, although the activation of wound-induced responses during Agrobacterium-mediated infection may contribute to uneven transgene expression, we detected consistently strong blue staining, reflecting successful *GUS* expression driven by this fragment of the *AhUBQ4* promoter. (Fig. 1B). Although the 973-bp and 2000-bp fragments showed differing levels of expression in leaves and stem discs, the differences were modest in both cases. Given the limited cargo capacity of vectors and the higher transformation efficiency of smaller constructs, we chose the 973-bp promoter fragment (*AhUBQ4pro* hereafter) as more suitable for practical use.

We performed RT-qPCR to quantify *GUS* expression levels using total RNA extracted from the infiltrated tissues. In *N. benthamiana*, the *AhUBQ4pro:GUS* construct resulted in significantly higher *GUS* expression than the *35S:GUS* construct, demonstrating its strong activity in transient assays (Fig. 1C). In peanut, the 35S promoter appeared to show slightly higher expression than that driven by the *AhUBQ4* promoter, although the difference did not reach statistical significance (Fig. 1C). Together, these results confirm that the *AhUBQ4* promoter is strong and constitutive, making it suitable for driving gene expression in model and peanut plants.

To evaluate the transcriptional activity of the *AhUBQ4* promoter relative to that of the commonly used 35S promoter, we placed the *GUS* and *Ruby* reporter genes under the control of each promoter; we then generated stable transgenic Arabidopsis lines and transgenic peanut hairy roots with these constructs. GUS staining in transgenic Arabidopsis seedlings revealed strong and uniform GUS activity driven by the *AhUBQ4* promoter across multiple independent transgenic lines (*AhUBQ4pro*:*GUS* #1, #2, and #3) (Fig. 1D). By contrast, seedlings transformed with the *35S:GUS* construct (*35S*:*GUS* #1, #2, and #3) exhibited weaker and more uneven GUS staining, particularly in roots, suggesting that the *AhUBQ4* promoter provides a more stable and consistent expression pattern than the 35S promoter in Arabidopsis. We obtained largely congruent results with the *Ruby* reporter (Fig. 1E). The peanut hairy roots transformed with the *AhUBQ4pro:Ruby* construct exhibited more intense and widespread red coloration, particularly in actively dividing root tips, compared with those transformed with the *35S:Ruby* construct, which resulted in less pigment accumulation.

RT-qPCR analysis confirmed these observations. We measured *GUS* expression in the leaves and roots of transgenic Arabidopsis seedlings from three independent lines (Fig. 1F). *AhUBQ4* promoter–driven lines showed significantly higher *GUS* transcript levels than 35S promoter–driven lines, with particularly strong expression in roots. Similarly, RT-qPCR analysis of *Ruby* expression in peanut hairy roots demonstrated that roots transformed with *AhUBQ4pro:Ruby* (*Ah* #1 and *Ah* #2) exhibited significantly higher *Ruby* transcript levels than those transformed with *35S:Ruby* (35S #1 and 35S #2) (Fig. 1G). These findings collectively demonstrate that the *AhUBQ4* promoter drives stronger and more stable gene expression than the 35S promoter.

Given the strong transcriptional activity of the *AhUBQ4* promoter toward its associated transgene, we evaluated its efficiency in mediating CRISPR/Cas9-based gene editing. To this end, we constructed a binary vector carrying two single guide RNAs (sgRNAs) targeting the peanut orthologs of Arabidopsis *HY5-HOMOLOG* (*HYH*) driven by the Arabidopsis *U6* promoter (*AtU6*) and a *Cas9-GFP* transgene under the control of the *AhUBQ4* promoter (Fig. 1H). As a control, we also used a construct with *Cas9-GFP* expressed from the 35S promoter. Fluorescence imaging of transgenic peanut hairy roots with a handheld light source revealed stronger GFP fluorescence when *GFP* was driven by the *AhUBQ4* promoter compared with *GFP* driven by the 35S promoter, confirming successful and robust transformation (Fig. 1I). Fluorescence stereomicroscopy imaging confirmed these observations, showing clear GFP fluorescence signals in positively transformed roots, with no detectable fluorescence in non-transformed controls (Fig. 1J). Moreover, the *AhUBQ4* promoter produced stronger GFP fluorescence than the 35S promoter.

To assess the efficiency of the 35S and *AhUBQ4* promoters in CRISPR/Cas9-mediated gene editing, we genotyped 15 transgenic and GFP-positive peanut hairy roots each following transformation with the 35S promoter–driven or *AhUBQ4* promoter–driven construct. On average, we detected 7.3 edits per transgenic root transformed with the *AhUBQ4* promoter–driven construct, and 5 edits in those transformed with the 35S promoter–driven construct (Fig. S1C). The *AhUBQ4* promoter thus appeared to exhibit higher editing efficiency, although the difference was not statistically significant. In addition, we analyzed sequencing chromatograms encompassing the target sites by categorizing them as “high-peak” or “low-peak” based on the height of the second-highest peak at the edited site. If the second peak exceeded half the height of the highest peak, we defined the chromatogram as high-peak; otherwise, we defined it as low-peak (Fig. S1D). For each sample, we looked at the 10 bp downstream of the edited site. Chromatograms with 5 or more high peaks were classified as high-peak, and those with fewer were considered low-peak. We evaluated a total of 15 transgenic GFP-positive roots per construct. At the *HYH* #1 site, the 35S promoter–driven construct showed slightly better results (seven vs. five high-peak chromatograms), but both promoters performed equally at the *HYH* #2 target site (Fig. S1E). In terms of types of mutations, the 35S promoter–driven construct primarily led to insertions and deletions, and the *AhUBQ4* promoter–driven construct also introduced substitutions at the sgRNA target sites (Fig. 1K).

The high editing efficiency and the presence of diverse mutation types confirm that the *AhUBQ4* promoter works well for CRISPR/Cas9-mediated genome editing in peanut hairy roots, supporting its utility for functional genomics in this system. This result, together with the strong, stable gene expression driven by the *AhUBQ4* promoter suggest that this promoter has potential to enhance transformation efficiency and transgene expression in peanut, making it a potentially useful tool for functional studies and gene editing.

## Materials and methods

### Identification and cloning of the *AhUBQ4* promoter in peanut

We identified all ubiquitin (UBQ) genes in the peanut genome by conducting BLAST searches against the Arachis hypogaea 'Tifrunner' reference genome available in public databases (https://dev.peanutbase.org/) (Bertioli et al. 2019), using all Arabidopsis UBQ genes as query sequences. Additionally, the public peanut transcriptome database (https://data.legumeinfo.org/Arachis/hypogaea/expression/Tifrunner.gnm2.ann2.expr.Tifrunner.Clevenger_2016/) was analyzed to assess the expression of *AhUBQ* genes across 15 different peanut tissues, aiming to identify the *AhUBQ* gene(s) with high and constitutive expression (Clevenger et al. 2016). Subsequently, the promoter region of the *AhUBQ4* gene, located upstream of the ATG start codon, was amplified from genomic DNA extracted from ‘Tifrunner’ leaves using the Hi-DNAsecure Plant Kit (DP350, TIANGEN, Beijing, China). The primers for promoter cloning are listed in Supplementary Table 3.

### Vector construction

The *AhUBQ4* promoter fragment was inserted into the pCAMBIA1381 vector, the pDR5-Ruby vector (He et al. 2020), and the CRISPR vector pJim19(Genta)-35S-Cas9 (Wang and Chen 2020) via homologous recombination using ClonExpress Ultra One Step Cloning Kit V3 (C117, Vazyme, Nanjing, China). Specifically, the *AhUBQ4* promoter was introduced into the pCAMBIA1381 vector via a multiple cloning site, in place of the *DR5* promoter in the pDR5-Ruby vector, and in place of the 35S promoter in the pJim19 (Genta)-35S-Cas9 vector.

For gene editing, the peanut homologs of Arabidopsis *HYH* (*HYHA* [arahy.8U3XLV]; *HYHB* [arahy.7JYX6X]) were selected as target genes. Because of the high sequence similarity between *HYHA* and *HYHB*, target sites were selected in the conserved regions shared by these two homologs. The sgRNA design and CRISPR vector construction followed the methods described by Wang and Chen (2020). The primers for vector construction are listed in Supplementary Table 3.

### Transient transformation

For transient transformation, *Nicotiana benthamiana* plants and peanut stem discs were used. Agrobacterium (*Agrobacterium tumefaciens*, strain GV3101) cultures harboring the desired expression vectors were used for infiltration into *N. benthamiana* leaves. A bacterial pellet derived from a culture of a positive colony was resuspended in infiltration buffer, consisting of liquid Murashige and Skoog (MS) medium containing 20 μg/L acetosyringone; the cell suspension was incubated at 28 °C for 1 h before use. The Agrobacterium cell suspension was then infiltrated into the abaxial side of *N. benthamiana* leaves, which were incubated in the dark for 24 h, before being exposed to light for 24 h. The leaves were then harvested, with one portion subjected to GUS staining and another stored at − 80 °C for gene expression analysis.

For transient expression in peanut, seedlings from the cultivar ‘Silihong’ were grown in darkness for 15 days. The upper and lower parts of the hypocotyl were sliced into stem discs of approximately 1 mm thick and immersed in the Agrobacterium cell suspension carrying the corresponding expression vector for 1 h. The infected explants were then placed in Petri dishes containing wet filter paper and incubated in the dark for 48 h. The samples were collected for analysis, with one set subjected to GUS staining and the other stored at − 80 °C for gene expression quantification.

### Stable transformation

Arabidopsis plants (accession Col-0) were transformed via the floral dip method (Clough and Bent 1998). Agrobacterium cultures harboring the expression vector of interest were incubated at 28 °C until they reached an OD_600_ of 0.8–1.0. T_1_ seeds were harvested from the dipped plants after 3–4 weeks, surface sterilized, and screened on MS medium containing 50 mg/mL Hygromycin (10,687,010, Thermo Fisher Scientific, CA, USA). For hairy root transformation, the corresponding expression construct was introduced into *Agrobacterium rhizogenes* strain K599. From peanut seedlings grown in tissue culture, 3–5-cm stem segments were excised and immersed in the infection solution and shaken at 120 rpm for 60–120 min. After infection, the *A. rhizogenes* solution was discarded, and the stem segments were washed 2–3 times with sterile water. The explants were then blotted dry on filter paper and placed on induction medium containing 200 mg/mL Timentin (116,238, Sigma-Aldrich, Saint Louis, MO 63103, USA), an *A. rhizogenes* bacteriostatic agent. The explants were maintained at 25 °C under a 16-h light/8-h dark photoperiod. Hairy root induction was typically observed after about 2 weeks.

### Reverse-transcription quantitative PCR (RT-qPCR) analysis of gene expression

For analysis of transient expression, the leaves from three independently infiltrated plants were pooled. For stable expression, tissues from six individual plants were combined. Each assay included three technical replicates. Total RNA was extracted using a TaKaRa MiniBEST Plant RNA Extraction Kit and reverse-transcribed with PrimeScript RT Master Mix. Specific primers (Supplementary Table 3) were designed using Primer3. Quantitative PCR (qPCR) was performed with TB Green Fast qPCR Mix on an ABI 7500 system in 20-μL reactions under the following conditions: 95 °C for 5 min, followed by 40 cycles of 95 °C for 30 s and 60 °C for 10 s. Relative expression levels were analyzed using the 2^−ΔΔCt^ method in Excel 2010, with statistical analysis and visualization conducted in GraphPad Prism 10. A *t*-test was used to assess the statistical significance of differences.

### GUS histochemical staining

Infiltrated tissues and corresponding controls (infiltrated with agrobacterium without a construct) were incubated in GUS staining solution (COOLABER, SL7160) at temperatures ranging from 25 °C to 37 °C, depending on tissue type and staining depth required. Specifically, *N. benthamiana* and Arabidopsis tissues were incubated at 37 °C for 5 h, and peanut stem discs and hairy roots were incubated at 25 °C for 8 h to allow sufficient substrate penetration. After staining, samples were cleared of chlorophyll in 70% (v/v) ethanol until no color was visible in control tissues.

### GFP visualization and detection of edited sites

GFP fluorescence in hairy roots was observed using a LUYOR-3415RG light source (LUYOR, USA) and a Leica fluorescence stereomicroscope (Leica Microsystems, Germany). To detect mutations at target sites, genomic DNA was extracted from 15 individual GFP-positive roots from 15 independent plants. The regions flanking the target sites were PCR-amplified using gene-specific primers and sequenced by Sanger sequencing to assess editing events. Chromatograms were classified as “high-peak” or “low-peak” based on whether the second-highest peak exceeded 50% of the highest peak at a putative edited site (Fig. S1D). Ten base pairs downstream of the edited site were evaluated; chromatograms with ≥ 5 high peaks were considered high-peak. Additionally, genomic DNA was extracted from pooled transgenic hairy roots derived from 10 explants. The regions flanking the target sites were amplified by PCR; the PCR amplicons were then purified using SanPrep Column DNA Gel Extraction Kit (NO. B518131, Sangon Biotech, Shanghai, China) and cloned into the pMD™ 18-T vector (Takara 6011). The plasmids extracted from 10 individual colonies were sequenced by Sanger sequencing to determine the types of mutation.

## Supplementary Information

Below is the link to the electronic supplementary material.

Supplementary file1 (DOCX 510 KB)

## Data Availability

Data will be made available on request.
