# Supplementary material for: The peanut Ubiquitin4 promoter drives stable gene overexpression and efficient multiplex CRISPR/Cas9 gene editing in peanut
Source: aBIOTECH. 2025 Jul 16;6(4):685–92. doi: 10.1007/s42994-025-00230-7 (PMC12647447; doi:10.1007/s42994-025-00230-7)
Supplement: Supplementary file 1 — Supplementary file1 (DOCX 510 KB) [file 42994_2025_230_MOESM1_ESM.docx]

**Supplementary Table 1.** FPKM values of peanut ubiquitin genes in different tissues.

| Gene ID | Leaf | Root | Flower | AerPeg | SubPeg | ExpPod | GynStlk | PodPt3 | PerPt5 | SdPt5 | PerPt6 | SdPt6 | SdPt7 | SdPt8 | SdPt10 |
| --- | --- | --- | --- | --- | --- | --- | --- | --- | --- | --- | --- | --- | --- | --- | --- |
| arahy.YREA25 | 5.13 | 3.64 | 3.64 | 4.06 | 3.32 | 5.48 | 1.92 | 6.35 | 4.10 | 8.38 | 3.26 | 7.93 | 6.08 | 5.80 | 3.57 |
| arahy.IIR79L | 63.71 | 93.17 | 10.97 | 156.97 | 293.04 | 168.07 | 26.20 | 85.79 | 82.63 | 252.23 | 74.61 | 222.98 | 151.78 | 71.15 | 49.46 |
| arahy.L94IR5 | 112.72 | 160.22 | 36.51 | 154.09 | 281.18 | 144.44 | 21.57 | 86.00 | 94.63 | 198.13 | 88.21 | 169.07 | 98.25 | 54.60 | 59.08 |
| arahy.U26HEC | 4.44 | 4.77 | 3.09 | 4.22 | 2.55 | 4.59 | 1.97 | 5.75 | 4.87 | 8.65 | 3.08 | 8.08 | 5.87 | 4.76 | 2.54 |
| arahy.077R6R | 179.95 | 218.01 | 40.45 | 482.28 | 703.60 | 525.09 | 140.29 | 364.02 | 305.91 | 501.39 | 286.19 | 364.01 | 275.88 | 174.25 | 159.65 |
| arahy.JV6JV5 | 120.78 | 192.30 | 259.76 | 303.94 | 376.25 | 335.71 | 427.76 | 465.10 | 377.16 | 253.78 | 423.23 | 280.39 | 241.99 | 109.67 | 58.81 |
| arahy.BR0PZ0 | 63.85 | 109.24 | 40.58 | 160.79 | 254.08 | 196.93 | 43.00 | 135.39 | 136.26 | 250.47 | 114.54 | 140.43 | 91.95 | 64.07 | 52.87 |
| arahy.WNC7YQ | 4.69 | 5.90 | 3.52 | 4.62 | 4.52 | 5.24 | 3.96 | 7.08 | 6.34 | 8.89 | 4.87 | 8.90 | 7.72 | 7.08 | 5.86 |
| arahy.P9J5FJ | 66.83 | 94.24 | 8.70 | 133.22 | 228.91 | 138.45 | 18.16 | 66.29 | 60.85 | 221.64 | 56.25 | 219.02 | 143.28 | 61.93 | 46.36 |
| arahy.I8M745 | 112.72 | 160.22 | 36.51 | 154.09 | 281.18 | 144.44 | 21.57 | 86.00 | 94.63 | 198.13 | 88.21 | 169.07 | 98.25 | 54.60 | 59.08 |
| arahy.AXB33C | 117.92 | 117.73 | 156.64 | 224.04 | 194.60 | 282.00 | 167.75 | 361.41 | 271.03 | 257.68 | 241.23 | 162.17 | 170.66 | 183.54 | 236.21 |
| arahy.M240R0 | 7.02 | 13.26 | 10.46 | 10.62 | 11.67 | 11.44 | 4.99 | 11.58 | 12.76 | 17.48 | 13.96 | 20.93 | 23.80 | 18.34 | 5.86 |
| arahy.E356RC | 1584.52 | 1890.51 | 3457.58 | 1978.68 | 1634.19 | 2116.64 | 2827.19 | 2476.57 | 2659.16 | 3283.13 | 3220.13 | 2435.50 | 1744.91 | 1038.93 | 435.10 |
| arahy.4WM679 | 71.74 | 113.44 | 39.45 | 300.96 | 1098.16 | 177.45 | 429.76 | 375.79 | 193.78 | 413.05 | 168.12 | 158.56 | 227.53 | 125.02 | 28.67 |
| arahy.N3UINY | 0.00 | 0.07 | 0.00 | 0.00 | 0.00 | 0.00 | 0.00 | 0.06 | 0.00 | 0.00 | 0.00 | 0.00 | 0.00 | 0.00 | 0.00 |
| arahy.KSY044 | 3.44 | 2.18 | 0.86 | 3.48 | 5.05 | 5.22 | 2.06 | 4.54 | 3.23 | 4.13 | 3.15 | 3.02 | 2.62 | 1.93 | 1.67 |
| arahy.U3BZ33 | 169.28 | 377.26 | 400.45 | 610.11 | 645.00 | 704.59 | 802.86 | 944.83 | 811.38 | 482.92 | 906.20 | 493.69 | 388.50 | 168.15 | 106.16 |
| arahy.387ULC | 20.81 | 46.72 | 14.92 | 50.70 | 89.18 | 58.40 | 7.64 | 37.36 | 38.62 | 75.10 | 35.95 | 58.57 | 22.20 | 9.48 | 8.48 |

**Supplementary Table 2.** Sequence of the *AhUBQ4* (arahy.E356RC) promoter (2000bp)

| GTTTGGCCACGCACGTAAGCTTGCTACTTGCTGTTGCTGCTCTGCTCTAAATATACATAGTACCATTCACTTTCCTCCTTAGCACCATGCCAGCATTGAGTTATCTATGACAAAGGTCGCTCATTTTTCATTTTTTCTCTCTTTTCATTAGTAGTGTTTATTGTTCAATATCTATCACCTAAAAGAAAAAAGGGTTCAATACAATTTTAAATTGTAATTACTTGAAAGTGATCAAAGGTTAGATAACTAAGTACTAGAAAATTATGTGGGAACTTTATCGATGGGGAGTTGCCAGGGGAATATTCTTAATGATTTATGATTTAGCTAAAATAACGCCTTAGCTCTTTCTCTCTTCGGGTTTCTCTCCCCGGGGGTACAAAAGAGTGAATATTTATGAAGTTTAATTTATTCACATTATAAGTAAGTGGAAAATTGAACATTAAACTTACATTTCTGTTGACTTTGACTAGTCAATGCTAAGTCGCATTACCAATTGATAGTTGTGGGAGAAAATTTTCAATTTAATAGTACCTTTTAATTGTCGGTAAAATTTGAAATATTCTATTTTGATCATTCAATTGAATTAAAAGCCTTTAATTTATGCTAATGGGTTATAATTCAAATGATATAGTTTTTTTATTCTTATCTAGGTGTTTTTAGTTTGAGTCTCATTTTTAATTTAAAAAATAAAAAAATAAAAACCTATAAATATAGTATTTCTCAATTTTTTCCTCCAAATTATTTTTTGGTGATAAACGACTTTAAAAAAAAAAGAAACAGAAGTGGATTCACTCAATGTTCTTTCCAGTTGATTATCCGATCCGTTTCTTTCAAGTTAGATCATAATTAGATAATAGATAATAATGTGCAACTGTGGATAAGCTTGAAAACTAAATGCCGGTTTTGCGTTCCTGATTAATAATCCTCAAAGAAAGAGTGATATAGAGAGTAGAGAGAGCAACACGCCAACATCTTTAATTTTGTAAAAAGAATTATCCACCAAATAAAAAATAGAATCTTTCTTTGCAGCCTCAGCTGCCGATTAATGGACAGTGGATCCCACCGTGATAATAAAATCTATAAAAGCCCACTTGTCTATTTTTTTATTTTGATTTTTGATTTGGTATACCGTATACCCCTTTCTCAGCCCACTTGCCAATTACTACAACTTGTGTGGGCCCAAATATTATGTTAAGGATCCACTTATAATAAGATAACATCTATTACCACAAAAAAAGAGATAATAACACCGTGGCGGTCAATACGAGAAGAGCGTTTGCGTGGTTTGTTGTTTGTTTGTGATGGGGAGGTCTGAGGTGCCTTAACTCCAACCAACGCCTTCCAATTTCCAACCCTTCACGTCTGGCCAAACATTGCCGTGTCTATAGTGTCAATTGACACCTCAACCGTACACGTGTCGCAAACTGATAAGGTAAATGGTCACCCTCTTTCTGTTGCGTAAATAAGGCGGACACCTCTTTATGCTATAAAGGAACCTCCAGACCCCATTGTTTCTTCACAATTCTCATTTTCATTCTCTCTTTGTTGTCCGAAATCCTTCAAGGTACCTTCCTCCTCTTTCTCTTTGCTTTTGATTCTGGTTCCCTTTAGTTACATCTGTAGGGTTTTGGCATTTCTTCTTAATCACCAATATTATCGGTTTTAGATTCGTTATATTATGTCTTTTGTTGCCTCGTTCCTTGGAAATTTTCTCTAGATCTGTTTATACTGCAATGATTTATGATATTTAATTGTTAAATCTGATCGGTTTTGATCACGCTTTTAAACTGCAATTTTTAGTTGCTTTCTGAAAGATCGAAGGCCTTTGTTTCGGATCGTAAATTTTCTGAAAAAATCTGAGATCAATTGTACCAATTTTGATTATAGGTTTTTTTTTTTTTTCCGTTGTTGATCCTGATTCATCTGTTTATGACGATGCGAATTTTAATGTACGTTTCCTTAACTAGTTTCTTATTTAATTGTTGTTAACGATGATGCAG |
| --- |

**Supplementary Table 3.** Primers used in this study

| Primer | Sequence (5′→3′) |
| --- | --- |
| Promoter cloning and vector construction | |
| pAhUBQ4-1000F | CCTCAGCTGCCGATTAATGGACA |
| pAhUBQ4-2000F | ATGGCCAAGTTTGGCCACGCA |
| pAhUBQ4-R | ATCTGCATCATCGTTAACAACAAT |
| pAhUBQ4-inGUS-1000F | TGGGCCCGGCGCGCCGAATTCCCTCAGCTGCCGATTAATGGA |
| pAhUBQ4-inGUS-2000F | TGGGCCCGGCGCGCCGAATTCATGGCCAAGTTTGGCCACGCA |
| pAhUBQ4-inGUS-R | TCGACGGATCCCCGGGAATTCATCTGCATCATCGTTAACAAC |
| pAhUBQ4-inRuby-F | ATAAGGGACTGACCACCCGGGCCTCAGCTGCCGATTAATG |
| pAhUBQ4-inRuby-R | CGAGGGTCGCATGATCCATATCTGCATCATCGTTAACAA |
| pAhUBQ4-inCas9-F | CAAGCTATCTAGAGCGGTACCCCTCAGCTGCCGATTAATGGACA |
| pAhUBQ4-inCas9-R | TTTAAAAGAAATGATTTAAATCTGCATCATCGTTAACAAC |
| sgRNA1-HYH-R | GATTGTTGCTGGGAAACGCCGCCG |
| sgRNA1-HYH-F | AAACCGGCGGCGTTTCCCAGCAAC |
| sgRNA2-HYH-R | GATTGGAATCAAGAGCTAAAGACT |
| sgRNA2-HYH-F | AAACAGTCTTTAGCTCTTGATTcc |
| RT-qPCR | |
| GUS-QF | CTGATAGCGCGTGACAAAAA |
| GUS-QR | GGCACAGCACATCAAAGAGA |
| Ruby-QF | CATCGGCAACATCCTTGAGG |
| Ruby-QR | GCCGGCTGTAACACTATTCG |
| AhELF1B-QF | AAGCTTCCCTGGCAAAGCTCAA |
| AhELF1B-QR | TTCCTCAGCTGCCTTCTTATCC |
| NtL25-QF | CCCCTCACCACAGAGTCTGC |
| NtL25-QR | AAGGGTGTTGTTGTCCTCAATCTT |
| Gene editing detection | |
| HYH-Cri-F1 | AAGATAGCGACGAAGATCTGTTC |
| HYH-Cri-R1 | GCGTGAAGGCAACTAATAACTATC |
| HYH-Cri-F2 | GAGGAACAGGGTTTCTGCCCAAC |
| HYH-Cri-R2 | TTGCGGAGCATGGTGTTCTCAT |

**
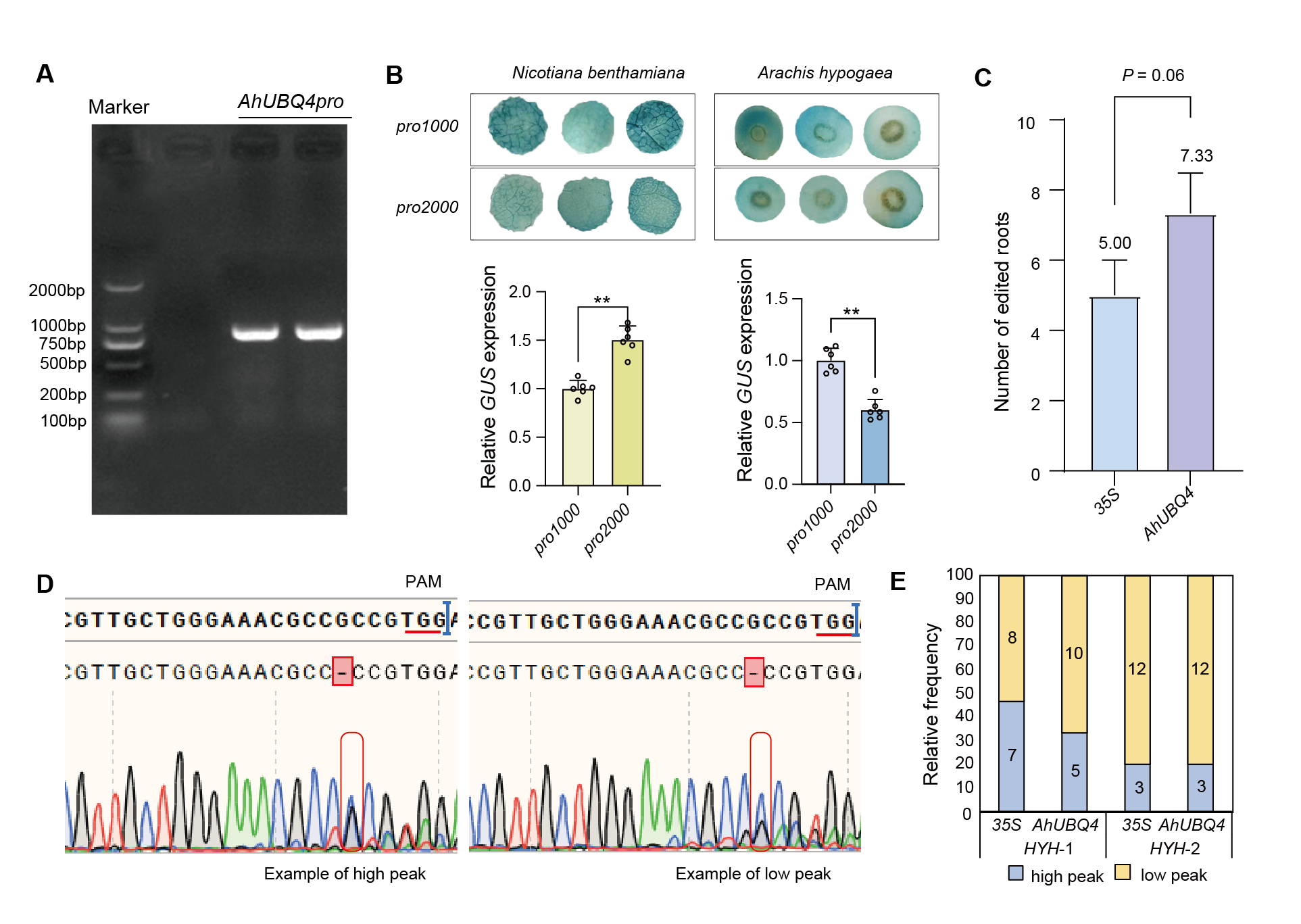
**

**Supplementary Fig. 1.** Functional characterization of the *AhUBQ4* promoter and editing efficiency in transgenic peanut roots.

**A** Agarose gel electrophoresis showing the PCR-amplified *AhUBQ4* promoter fragment (~973 bp) from peanut genomic DNA. **B** GUS staining and relative *GUS* expression in *Nicotiana benthamiana* leaves and peanut stem discs infiltrated with *Agrobacterium tumefaciens* cultures harboring constructs driven by a 973-bp or 2000-bp fragment of the *AhUBQ4* promoter (*pro1000* and *pro2000*, respectively). Values are means ± standard deviation (SD; **, *P* < 0.01, two-tailed Student’s *t*-test). **C** Average number of edited roots out of 15 GFP-positive transgenic roots from plants transformed with CaMV 35S promoter–driven or *AhUBQ4* promoter–driven *Cas9* constructs. Values are means ± SD (*P* = 0.06, two-tailed Student’s *t*-test). **D** Representative Sanger sequencing chromatograms showing examples of high-peak and low-peak patterns, based on the height of the second-highest base signal near the editing site. PAM, protospacer-adjacent motif. **E** Quantification of high-peak and low-peak chromatograms at two single guide RNA (sgRNA) target sites (*HYH-1* and *HYH-2*) among 15 transgenic roots per group.
